# Impact of the diagnostic delay of acromegaly on bone health: data from a real life and long term follow-up experience

**DOI:** 10.1007/s11102-022-01266-4

**Published:** 2022-08-03

**Authors:** Sabrina Chiloiro, Antonella Giampietro, Irene Gagliardi, Marta Bondanelli, Miriam Veleno, Maria Rosaria Ambrosio, Maria Chiara Zatelli, Alfredo Pontecorvi, Andrea Giustina, Laura De Marinis, Antonio Bianchi

**Affiliations:** 1 Endocrinology and Diabetology Department, Fondazione Policlinico Universitario A. Gemelli, IRCCS, Rome, Italy; 2 Department of Translational Medicine and Surgery, Università Cattolica del Sacro Cuore, Rome, Italy; 3 Section of Endocrinology, Geriatrics & Internal Medicine, Department of Medical Sciences, University of Ferrara, Ferrara, Italy; 4 Institute of Endocrine and Metabolic Sciences, San Raffaele, Vita-Salute University and IRCCS Hospital, Milan, Italy

**Keywords:** Vertebral fractures, Skeletal fragility, GH secreting adenoma, GH, IGF-1, Osteomalacia

## Abstract

**Introduction:**

Acromegaly is a chronic disease with systemic complications. Disease onset is insidious and consequently typically burdened by diagnostic delay. A longer diagnostic delay induces more frequently cardiovascular, respiratory, metabolic, neuropsychiatric and musculoskeletal comorbidities. No data are available on the effect of diagnostic delay on skeletal fragility. We aimed to evaluate the effect of diagnostic delay on the frequency of incident and prevalent of vertebral fractures (i-VFs and p-VFs) in a large cohort of acromegaly patients.

**Patients and methods:**

A longitudinal, retrospective and multicenter study was conducted on 172 acromegaly patients.

**Results:**

Median diagnostic delay and duration of follow-up were respectively 10 years (IQR: 6) and 10 years (IQR: 8). P-VFs were observed in 18.6% and i-VFs occurred in 34.3% of patients. The median estimated diagnostic delay was longer in patients with i-VFs (median: 11 years, IQR: 3), in comparison to those without i-VFs (median: 8 years, IQR: 7; p = 0.02). Age at acromegaly diagnosis and at last follow-up were higher in patients with i-VFs, with respect to those without i-VFs. The age at acromegaly diagnosis was positively associated with the diagnostic delay (p < 0.001, r = 0.216). A longer history of active acromegaly was associated with a high frequency of i-VFs (p = 0.03). The logistic regression confirmed that patients with a diagnostic delay > 10 years had 1.5-folds increased risk of developing i-VFs (OR: 1.5; 95%CI: 1.1–2; p = 0.017).

**Conclusion:**

Our data showed that the diagnostic delay in acromegaly has a significant impact on VF risk, further supporting the clinical relevance of an early acromegaly diagnosis.

**Supplementary Information:**

The online version contains supplementary material available at 10.1007/s11102-022-01266-4.

## Introduction

Acromegaly is a clinical syndrome characterized by growth hormone (GH) and insulin-like growth factor-1 (IGF-1) excess. Chronic excess of GH and IGF-1 induces progressive somatic changes and is associated with multiple complications as cardiovascular, respiratory, metabolic, neoplastic, musculoskeletal disease, that are responsible for increased mortality and compromised quality of life [1–3]. The musculoskeletal disease in acromegaly includes secondary osteoarthritis and skeletal fragility with an increased risk of vertebral fractures (VF) [4]. VFs involve around 25–40% of acromegaly patients [5]. Several studies investigated the risk factors for the occurrence of VFs, identifying as the most significant the higher levels of GH and IGF-1, the longer length of biochemically active disease and the presence of pre-existing VFs [2, 4–10].

As the clinical features of acromegaly develop insidiously, the diagnosis is often significantly delayed [11]. The diagnostic delay in acromegaly ranges from 2 to 25 years [12, 13]. Data from the Swedish National Patient Registry on 603 acromegaly patients showed that 23% of patients had a diagnostic delay from 1 to 5 years, the 17% of patients had a diagnostic delay from 5 to 10 years and up to 24% of patients had a diagnostic delay over than 10 years [14]. Moreover, patients with a longer diagnostic delay were affected more frequently by cardiovascular, respiratory, metabolic, neuropsychiatric and musculoskeletal comorbidities. An early diagnosis and a proper treatment can prevent the development of irreversible complications of the disease, improving in parallel the patients’ quality of the life [15].

Since data on the impact of the diagnostic delay on the occurrence of VFs in acromegaly are not yet available, we aimed to investigate the effect of the diagnostic delay on the prevalence and incidence of VFs, in a multicenter and retrospective large cohort of acromegaly patients.

## Patients and methods

A longitudinal, retrospective, observational and multicenter study was performed on acromegaly patients. All patients were consecutively enrolled according to the following inclusion and exclusion criteria.

Inclusion criteria were

1. diagnosis of acromegaly;
2. age older than 18 years;
3. patients attending out-patient Pituitary clinics;
4. availability of sequential spine X-ray evaluations at least at acromegaly diagnosis and at last evaluation visit;
5. last endocrine evaluation conducted within the last 12 months.

The exclusion criteria were:

1. diagnosis of active neoplastic disease;
2. diagnosis of primary hyperparathyroidism and MEN-1 syndrome;
3. untreated hyperthyroidism
4. use of bone active drugs (except calcium and vitamin D) in the 12 months prior to study entry (acromegaly diagnosis);
5. treatment with drugs known to cause fragility fractures (16) with the exception of glucocorticoid replacement therapy for hypopituitarism;
6. history of spine surgery.

### Objectives

The primary objective of the study was to evaluate the association between the diagnostic delay in acromegaly and both the prevalence of VFs at acromegaly diagnosis and the incidence of VFs, during the follow-up.

As secondary objectives, we evaluated the correlations between incident VFs and (1) activity of acromegaly at the end of follow-up, (2) duration of active disease, (3) IGF-1 and GH values during follow-up, (4) prevalent VFs at the acromegaly diagnosis, (6) hypopituitarism status, (7) patients’ gender, (8) treatment for osteoporosis and acromegaly.

All the clinical information were retrospectively collected by the medical records of the patients.

### Evaluation of acromegaly

Acromegaly was diagnosed according to guidelines available at the time of the first observation of the patients. During the follow-up, acromegaly patients underwent periodical evaluation of GH and IGF-1, to define the control of the disease. According to disease status, patients were classified as cured, controlled or affected by active disease. Acromegaly was defined:

- cured in patients off-therapy for at least six consecutive months with normal age and gender-adjusted IGF-1 values and random/integrate GH was below 1.0 ng/mL and with GH nadir < 0.4 ng/mL during oral glucose tolerance test (OGTT) in not-diabetic patients;
- controlled in patients treated with medical therapy, with normal age and gender-adjusted IGF-1 values and random GH was below 1.0 ng/mL [17];
- active in patients treated with medical therapy, IGF-1 concentrations above the normal ranges for age and gender, and with random GH higher than 1.0 ng/mL [18].

Patients on treatment with Peg-V were evaluated only by serum IGF-1 [18]. IGF-1 was expressed according to upper limit of normal (ULN), based on normative data for each center laboratory.

According to the consensus of acromegaly [17], during the follow-up, the IGF-1 levels reflect the clinical activity of the disease. Serum GH levels can be used to assess the control. In patients with discrepant levels of GH and IGF-1, the consensus recommend relying on IGF-1 values, after making sure on the use of well-validated IGF-1 assay and after ruling out pre-analytic and analytical confounding factors [17], such as malnutrition, obesity, eating disorders, poorly controlled diabetes mellitus, cystic fibrosis, hepatic and renal disease,, hypothyroidism, hyperthyroidism, sepsis, supraphysiological testosterone replacement [19].

The diagnostic delay was defined as the number of years between the time of ascertained acromegaly diagnosis and the presumed time of occurrence of the first acromegaly related symptoms. The length of active acromegaly was estimated as the number of months between the time of reaching two consecutive values of IGF-1 within the normal ranges for age and gender and the time of acromegaly diagnosis.

### Definition of diagnostic delay

**Fig. 1:**
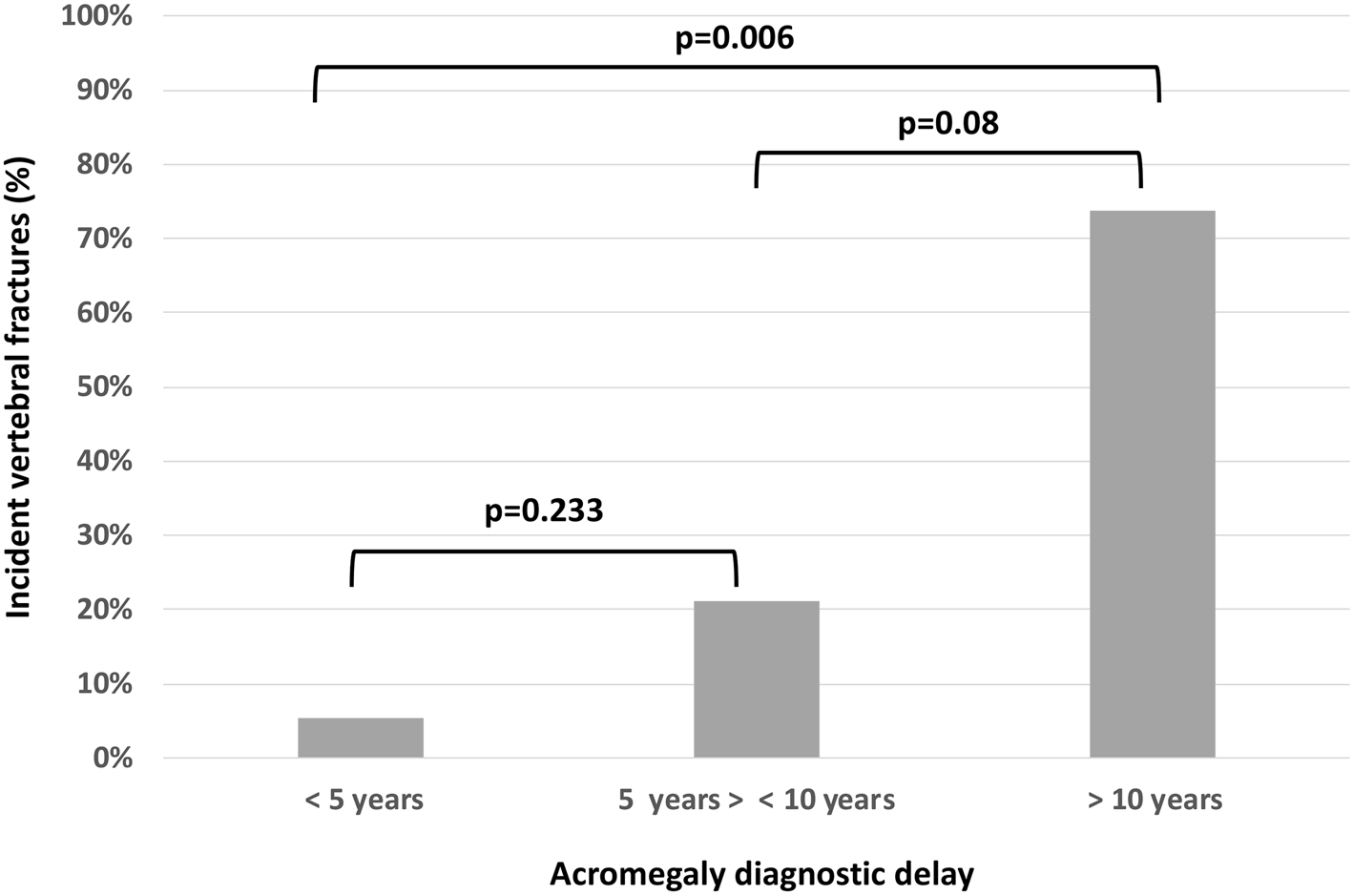
Histogram showing the percentage of incident vertebral fractures according to diagnostic delay. Univariate analysis

The diagnostic delay was calculated as the elapsed time between the date of the first reported comorbidity and the date of acromegaly diagnosis, as recently reported by Esposito et al. [14]. The date of diagnosis was defined as the first specialized healthcare visit or admission with acromegaly diagnosis. The date of onset of the first comorbidity was defined as the first registration of any predefined comorbidity from medical records. The more specific acromegaly associated comorbidities were taken in account, such as visual-field defects, headache, hypopituitarism, arthropathy, osteoporosis, vertebral fractures, non-traumatic fractures, carpal tunnel syndrome, macroglossia, hypertension, cardiomyopathy, cardiac hypertrophy/heart disease, heart failure, cardiac dysrhythmia, ischemic heart disease, sleep apnea, diabetes, impaired glucose tolerance, second benign and malign tumors, nodular thyroid disease, polyps of colon and of vocal cord and larynx [14] (see Fig. 1).

### Evaluation of vertebral fractures

Prevalent and incident vertebral fractures (VFs) were investigated respectively on spine X-rays performed at acromegaly diagnosis and at last visit, using quantitative morphometric approach, as previously described [6]. Anterior (Ha), middle (Hm), and posterior (Hp) vertebral heights were measured and height ratios were calculated for each vertebra from T4 to L4. Prevalent VFs were identified on the baseline radiographs, whereas incident VFs were identified on spinal radiographs obtained at the follow-up, and they were defined as a decrease of 20% or more and at least 4 mm in length in any of the three vertebral heights (Ha, Hm, or Hp) compared with the baseline radiograph. As for clinical practise at our Pituitary divisions and according to 2013 guidelines on the management of comorbidities in acromegaly [20], vertebral morphometry on thoracic and lumbar spine x-ray is conducted at the time of acromegaly diagnosis and every two years, during follow-up.

### Statistical analysis

The patients’ cohort was described in its clinical and demographic features using descriptive statistics techniques. Normality of continuous variables was checked using Kolmogorov–Smirnov test. Quantitative variables were expressed as median and range and qualitative variables as absolute and percentage frequency. Chi square test (or Fisher exact test when necessary) and Mann Whitney non-parametric tests were used to compare categorical and quantitative un-paired data. The variables that reached the statistical significance at the univariate analysis entered the logistic regression. The analyses were performed using SPSS software version 24.0 for Windows.

## Results

A total of 172 patients entered the study, from an initial cohort of 187 cases (10 cases were excluded for the absence of sequential spine X-ray evaluations and 5 patients were excluded as considered lost at follow-up, as last evaluation was conducted over 12 months).

Among the study cohort, 91 patients were females (52.9%). Median age at ACRO-diagnosis was 42.5 years (IQR: 19). Median estimated diagnostic delay was of 10 years (IQR: 6).

Thirty-two patients were bearing VFs already at ACRO-diagnosis (18.6%). Among the 140 patients without VFs at ACRO-diagnosis, 48 patients developed new vertebral fractures (34.3% of cases p < 0.001) during follow-up. Among the 32 patients with prevalent VFs, 15 patients developed new incident VFs during the follow-up (46.9% of cases). Median duration of follow-up was 10 years (IQR: 8). During follow-up, 121 patients (70.3%) received vitamin D3 and consequently none case of secondary hyperparathyroidism was detected. Doses of vitamin D ranges from 500 to 3300 units per day; ten patients (5.5%) underwent treatment with bone active drugs.

At last follow-up visit, fifty-five patients (31.9%) were considered cured after pituitary surgery and/or after six consecutive months from the discontinuation of medical therapy. One-hundred three patients (59.9%) were considered controlled during medical therapies and 14 patients (8.2%) were considered affected by active acromegaly. In this study cohort, five patients were considered affected by active acromegaly with discordant GH and IGF-1 levels. These patients carried in at least two consecutive hormonal assessments random GH < 1 ng/mL and IGF-1 upper the age and gender-adjusted range of normality. After ruling out the causes of discrepant levels of GH and IGF-1 (such as physical exercise, pregnancy, hypothyroidism and hyperthyroidism) [19], the patients were considered affected by active acromegaly, also in accordance with the persistence of symptoms of active acromegaly.

### Vertebral fractures at acromegaly diagnosis

As showed in Table 1, among the whole study population, the prevalence of vertebral fractures at ACRO-diagnosis did not differ according to gender, age, GH and IGF-1 value at acromegaly diagnosis, smoking, alcohol abuse, gonadal function, central hypoadrenalism and dosage of hydrocortisone or equivalent corticosteroids.

**Table 1:** Skeletal fragility at acromegaly diagnosis and during follow-up. §: 140 patients that were not diagnosed for skeletal fragility at the time of acromegaly diagnosis, *: among 100 patients without skeletal fragility at acromegaly diagnosis and on medical treatment at the end of the study

	Prevalent vertebral fractures at ACRO-diagnosis			Incident vertebral fractures during follow-up (§)		
	Yes	No	p-value	Yes	No	p-value
Age at ACRO diagnosis median, (IQR)	41 (20)	47 (15)	0.87	45 (22)	38 (20)	0.04
Estimated diagnostic delay median years, (IQR)	10.5 (10.5)	10 (6)	0.57	12 (2)	9 (7)	0.02
Gender						
Females n, (%)	19 (59.4%)	72 (51.4%)	0.42	30 (62.5%)	50 (54.3%)	0.06
Males n, (%)	13 (40.6%)	68 (48.6%)		18 (37.5%)	42 (45.7%)	
GH at ACRO diagnosis median, (IQR)	4 (7)	6 (8)	0.43	6 (7)	3.4 (7)	0.07
IGF-I x ULN at ACRO diagnosis median, (IQR)	2.7 (0.5)	2.4 (1.5)	0.37	2.3 (1.4)	2.4 (2)	0.67
Central hypoadrenalism						
Yes n, (%)	14 (43.8%)	55 (39.3%)	0.62	19 (39.6%)	36 (39.1%)	0.96
No n, (%)	18 (56.3%)	85 (60.7%)		29 (60.4%)	56 (60.9%)	
Dosage of hydrocortisone (or equivalent)						
Patient treated with ≤ 20 mg/daily n, (%)	10 (71.4%)	36 (65.5%)	0.67	13 (68.4%)	23 (63.9%)	0.74
Patient treated with > 20 mg/daily n, (%)	4 (28.6%)	19 (34.5%)		6 (31.6%)	13 (36.1%)	
Gonadal function						
Normal n, (%)	25 (78.1%)	105(75%)	Ref	32(66.7%)	73(79.3%)	Ref
Treated central hypogonadism n, (%)	0 (0%)	0 (0%)	Na	3 (6.3%)	15(16.3%)	0.23
Untreated central hypogonadism n, (%)	4 (18.2%)	21(15%)	0.9	2 (4.2%)	1 (1.1%)	0.18
Menopause n, (%)	3 (9.4%)	14(10%)	0.8	11(22.9%)	3(3.3%)	0.001
Acromegaly outcome						
Cured/controlled n, (%)	Na	Na	Na	45 (93.8%)	83 (90.2%)	0.48
Active n, (%)				3 (6.3%)	9 (9.8%)	
GH at follow-up median, ng/mL (IQR)	Na	Na	Na	1 (1.3)	0.6 (2)	0.8
IGF-I x ULN at follow-up median, (IQR)	Na	Na	Na	0.6 (1.4)	0.8 (0.3)	0.15
Length of active acromegaly median months, (IQR)	Na	Na	Na	60 (58)	36 (56)	0.03
Medical treatment (*)						
First generation somatostatin analogues n, (%)	Na	Na	Na	26 (74.3%)	38 (60.3%)	Ref
Dopamine agonist n, (%)	Na	Na		2 (5.7%)	2 (3.2%)	0.24
Pegvisomant n, (%)	Na	Na		6 (17.1%)	20 (31.7%)	0.11
Pasireotide Lar n, (%)	Na	Na		2 (5.7%)	2 (3.2%)	0.24
Pasireotide Lar plus Pegvisomant n, (%)	Na	Na		1 (2.9%)	1 (1.6%)	0.27
Bone active drugs, n (%)	Na	Na	Na	3 (30%)	7 (70%)	0.589

### Vertebral fractures during follow-up

**Fig. 2:**
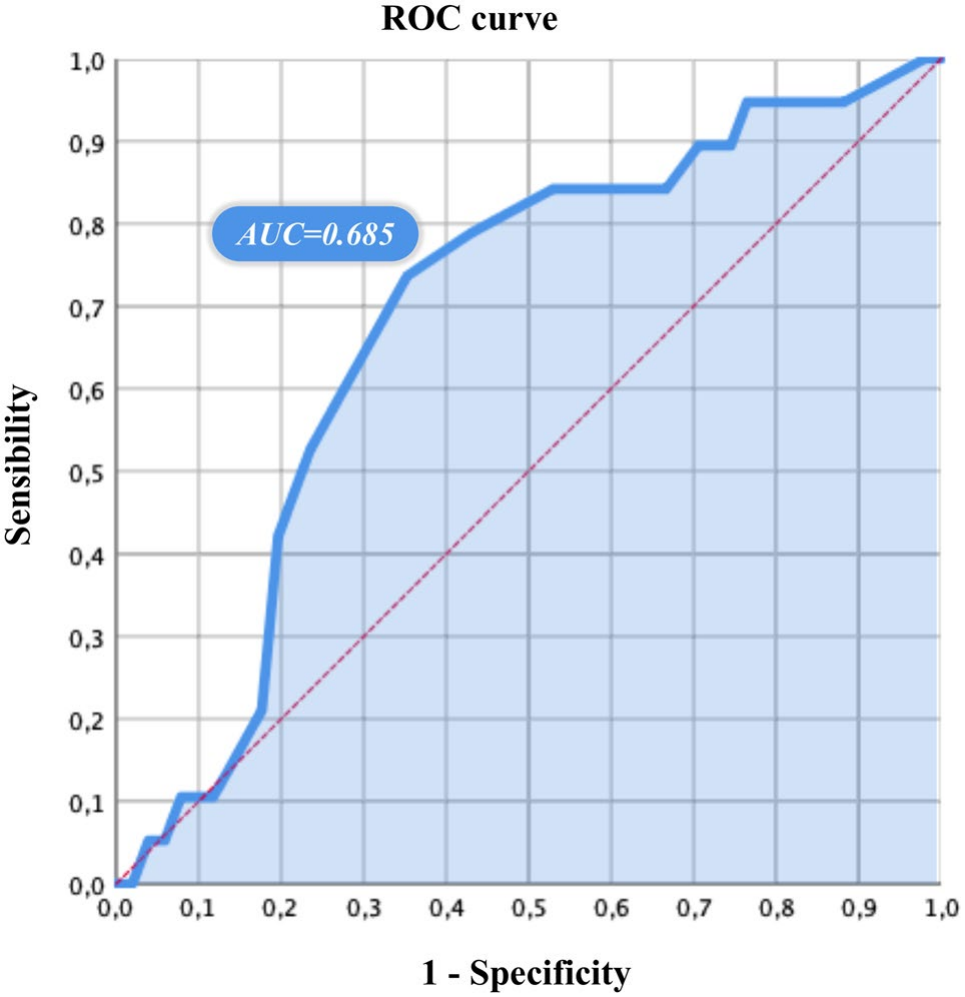
Roc Curve. The area under the ROC curve developed for the month of diagnostic delay in patients who developed incident vertebral fractures was was 0.68 (95% CI 0.5–0.82; p = 0.04), as showed in Fig. 2. Optimal cut-off was identified at 10 years (specificity: 90.9% sensitivity: 74%)

As showed in Table 1, the occurrence of incident vertebral fractures associated with the diagnostic delay. In fact, the median estimated diagnostic delay was significantly longer in patients with incident VFs (median 11 years, IQR: 3), in comparison to the diagnostic delay evaluated for patients without.

incident VFs (median 8 years, IQR: 7; p = 0.02). As showed in Fig. 2, the incidence of VFs progressively increased with a longer diagnostic delay: incidental VFs occurred in 5.3% of patients with a diagnostic delay shorter than 5 years, in 21.1% of patients with a diagnostic delay shorter than 10 years and in 73.7% of patients with a diagnostic delay longer than 10 years (p = 0.01). The area under the ROC curve developed for the estimated diagnostic delay was 0.68 (95% CI 0.5–0.82; p = 0.04), as showed in Fig. 2. Optimal cut-off value of diagnostic delay for predicting the risk of incident vertebral fractures was over 10 years (specificity: 90.9% sensitivity: 74%).

**Fig. 3:**
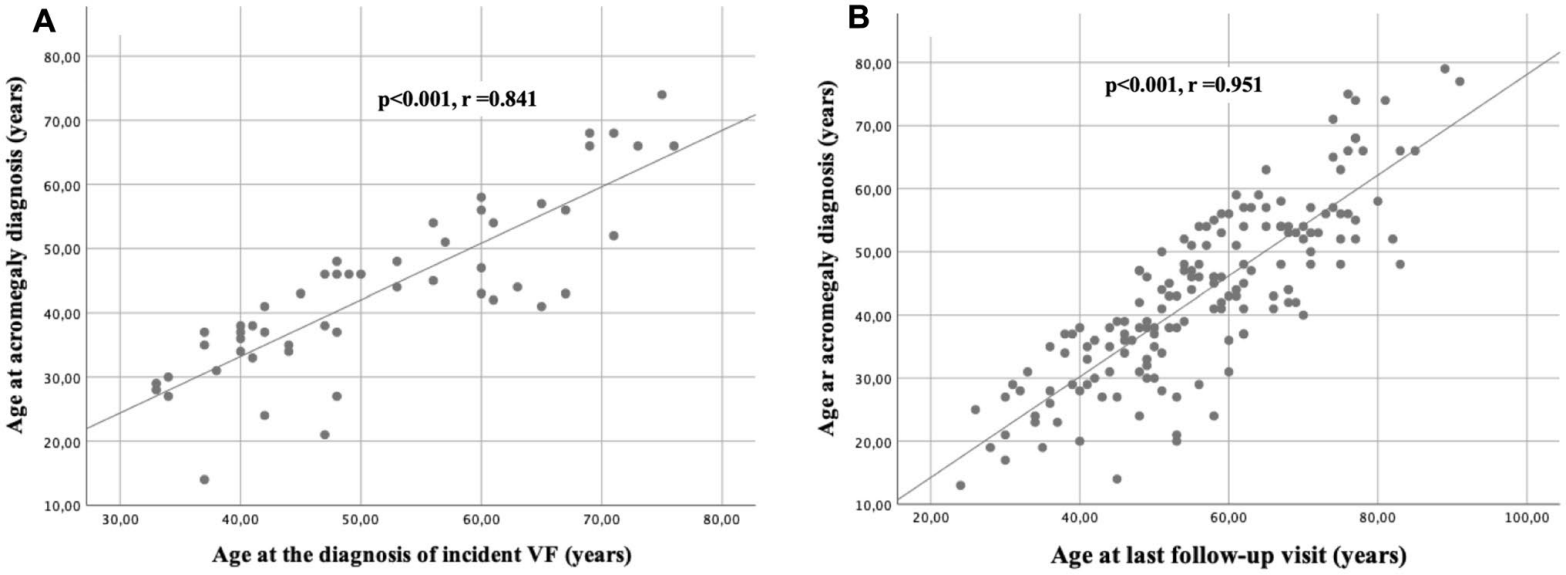
Scatter plot correlating in **a** the age of patients at the acromegaly diagnosis and at the time of the diagnosis of incident VFs (p < 0.001, r = 0.841) and in **b** the age of patients at the acromegaly diagnosis and the age of patients at last follow-up (p < 0.001, r = 0.951)

**Fig. 4:**
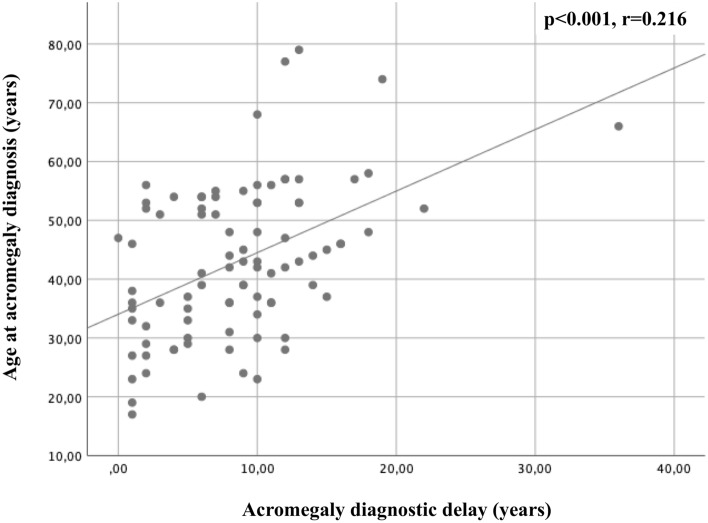
Scatter plot correlating age at acromegaly diagnosis and diagnostic delay (p < 0.001, r = 0.216)

We found that patients who experienced incident VFs were older than those without VFs both at the time of acromegaly diagnosis, both at the time of the diagnosis of incident VFs and, at last follow-up. Anyway, we found a positive correlation between the age of patients at the acromegaly diagnosis and at the time of the diagnosis of incident VFs (p < 0.001, r = 0.841) and between the age of patients at the acromegaly diagnosis and the age of patients at last follow-up (p < 0.001, r = 0.951), as showed in Fig. 3. In particular, the median age at acromegaly diagnosis was 45 years (IQR: 22) in patients with incident VFs and of 38 years (IQR: 20 p = 0.04) in patients without incident VFs. Interestingly, we found a positive correlation between the age at acromegaly diagnosis and the diagnostic delay (p < 0.001, r = 0.216), as showed in Fig. 4. Moreover, we found that patients with incident VFs were older (median age at last follow-up 61.3 years IQR: 13) as compared to patients without incident VFs (median age at last follow-up 52 years IQR: 13, p < 0.001), also at last follow-up. A positive correlation between age at last follow-up and diagnostic delay was identified (p < 0.001, r = 0.466). No gender difference in the incidence and prevalence of VFs was observed.

A longer length of active acromegaly was associated with the development of VFs during follow-up (p = 0.03): median duration of active acromegaly was 60 months (IQR: 58) in patients with incident VFs and 36 months in patients without VFs (IQR: 56; p = 0.03).

### Logistic regression

Age at acromegaly diagnosis, at the time of diagnosis of incident VFs and at last follow-up in the whole cohort and in females, diagnostic delay and length of active acromegaly entered the logistic regression. As shown in Table 2, the logistic regression showed that the only determinant for the event incident vertebral fractures was the diagnostic delay longer than 10 years. Patients with a diagnostic delay over 10 years had a 1.5-fold increased risk for developing incident VFs (OR: 1.5; 95%CI: 1.1–2; p = 0.017).

**Table 2:** Logistic regression for the occurrence of incident vertebral fractures during acromegaly follow-up

	p-value	OR (95%CI)
Age at acromegaly diagnosis	0.676	Na
Menopause	0.99	Na
Diagnostic delay	0.008	1.5 (1.1–2)
Length of active acromegaly	0.428	Na
Age at the diagnosis of incident VFs	0.432	Na
Age at last follow-up	0.175	Na

## Discussion

The diagnostic delay in acromegaly has recently emerged as a relevant clinical issue, resulting in increased mortality and frequency of metabolic, musculoskeletal and cardiovascular comorbidities [21].

In this study, we describe the relevant impact of the diagnostic delay on acromegalic osteopathy in a retrospective and longitudinal cohort of 172 patients, in whom were analysed both the prevalence of VFs at acromegaly diagnosis and the incidence during a follow-up of 10 years.

In this multi-center cohort, we found a prevalence of VFs of 18.6% and an incidence of 34.3%. These data are in line with previous studies that reported a VF’s prevalence ranging from 32 to 42% and an incidence ranging from 26 to 35% [7, 22–26].

The determinants of skeletal fragility in acromegaly are not yet completely clarified, despite several published studies. Until now, high levels of GH and IGF-1, the persistence of active acromegaly despite therapies, the untreated hypogonadism, the overtreated central hypoadrenalism were recognized as risk factors for skeletal fragility in acromegaly [7, 20–27].

It is well known that the GH and the IGF-1 act as regulators of the bone homeostasis. GH promotes the osteoblastogenesis and the chondrogenesis and inhibits the adipogenesis. GH stimulates also, either directly or indirectly through IGF-1, the function of mature osteoblast, through the carboxylation of osteocalcin, which impairs osteoclastogenesis [28, 29]. On mature osteoblasts, IGF-1 up-regulates type I collagen transcription and decreases the synthesis of matrix metalloproteinase 13, a collagen-degrading protease [30]. Indirectly, IGF-1 promotes osteoblastogenesis by the stabilization of the β-catenin, a signaling molecule of the wnt canonical signaling pathway, which is essential for osteoblastogenesis [31, 32]. The function of IGF-1 on osteoclasts is less clear: IGF-1 may induce osteoclastogenesis through the ligand of the activator receptor of nuclear factor kB (RANK-L), although this effect is tempered by the production of osteoprotegerin mediated by GH [33, 34].

A longer time of exposure to GH and IGF-1 hypersecretion was also associated to skeletal fragility in acromegaly and to an increased risk of VFs. The long term exposure to GH and IGF-1 in acromegaly may be due both to the persistence of active disease and both to the diagnostic delay.

Taking into account the impact of GH and IGF-1 hypersecretion on the occurrence of systemic complications (as cardiovascular, respiratory, metabolic, neoplastic and musculoskeletal ones), several studies had underlined the importance of an early achievement of normalization of GH and IGF-1 levels. Recently, the longer diagnostic delay was recognized as a risk factor for the occurrence on musculoskeletal, cardiovascular, respiratory, metabolic, oncological and neuropsychiatric comorbidities in acromegaly [14].

In parallel, our study discloses that a long diagnostic delay is a clinical significant risk factor for the occurrence of skeletal fragility and VFs. We found that the occurrence of incident VFs during follow-up was more frequent in acromegaly patients with a longer diagnostic delay. Our data can suggest a cumulative effect over time of the GH and IGF-1 hypersecretion on bone homeostasis, possibly explaining the effect of acromegaly diagnostic delay on the incidence of VFs rather than on their prevalence. In fact, to our knowledge, this study described for the first time that a diagnostic delay longer than 10 years is associated with an increased incidence of VFs, which were previously reported to be associated to a reduced quality of life and to the presence of hyperkyphosis [35, 36]. In fact, thoracic hyperkyphosis may impair the cardiorespiratory function and predispose to worse outcome of pulmonary infections such as COVID-19 [37].

In addition, we found that patients without VFs at acromegaly diagnosis had a low frequency of incident vertebral fractures during the follow-up. Conversely, patients who carried VFs at acromegaly diagnosis had a 1.5 fold increased risk for the occurrence of new vertebral fractures during the follow-up*.* These data suggest that the skeletal fragility may be considered a relatively early complication of acromegaly disease, confirming data reported at the time of neurosurgery [8]*.*

The early onset and the irreversibility of VFs requires strict screening and surveillance protocols since the time of acromegaly diagnosis [38, 39], that includes the assay of markers of bone formation and resorption, calcium, vitamin D and parathyroid hormone (PTH), the vertebral morphometry and possibly the analysis of bone microstructure though the trabecular bone score (TBS) and the microindentation technique [40]. In addition, the vitamin D supplementation and the prescription of bone active drugs may improve the bone quality in acromegaly patients, in particular in those with active disease, together with the early normalization of GH and IGF-1 levels [25].

Despite VFs are rarely diagnosed clinically in acromegaly patients for their subclinical and not-specific onset symptoms (such as back pain) [41], VFs are considered a clinical relevant issue. VFs reflect the bone health. The occurrence of VFs affects the quality of life and the overall survival of the patients [35–37]. For all these reasons, according to 2013 and 2020 guidelines on the management of comorbidities in acromegaly patients [20, 39], we routinely performed in our clinical practise a spine X-ray with morphometry, at acromegaly diagnosis and every two years, during the follow-up. Interestingly, the last guidelines on 2020 for acromegaly comorbidities suggested an annual screening with vertebral morphometry on thoracic and lumbar spine x-ray in acromegaly patients at high risk for the occurrence of VFs, as those with history of non-traumatic or vertebral fractures, osteopenia/osteoporosis, kyphosis, untreated hypogonadism and biochemical active acromegaly [39]. The vertebral morphometric approach has been widely recognized as the “gold standard” for the evaluation of bone health in acromegaly [9]. The dual-energy X-ray absorptiometry (DXA) leads to inconsistent densitometry results, for the variable distribution of trabecular and cortical bones in the different skeletal sites, for the abnormalities of bone structures, osteophytes, face-joint hypertrophy, increased periosteal ossification and bone enlargement in acromegaly [10]. More recently, if the spine X-ray is not available, the morphometry on chest X-ray has been proposed as a possible alternative for the diagnosis of VFs [8].

Our data showed moreover that the incidence of VFs was significantly higher in patients older both at the time of acromegaly diagnosis, at the time of diagnosis of incident VFs and at last follow-up. In an interesting way, we found a positive correlation (as showed in Fig. 3) between the age at acromegaly diagnosis and the age at the diagnosis of incident VFs, but also between the age at acromegaly diagnosis and at the age of the last follow-up visit, suggesting that the duration of follow-up is not dependent from the age of patients at the diagnosis of acromegaly (p = 0.399) and as for consequence from the age of patients in starting cures and follow-up.

These data suggest that also the physiological process of aging may play a crucial role in the occurrence of VFs in older patients with acromegaly [42]. Moreover, in a recent case–control study, acromegaly patients aged over 65 years old showed more frequently musculoskeletal and bone diseases than no-acromegaly matched group (52% vs. 12%; 64% vs. 10%; P < 0.05) supporting that aging and acromegaly could affect both negatively bone health [43]. Osteoporosis is considered the most common metabolic disease in the elders with a prevalence of around 39% in study population in elders in 2020 in Spain and China [44]. Senile osteoporosis is a multifactorial disease, with a central role played by the high levels of PTH. In elderly subjects, hyperparathyroidism may be due to vitamin D deficiency [45] and to the chronic kidney disease. In fact, serum PTH values generally increase when estimated glomerular filtration rate falls below 60 mL/min/1.73 m2 [46].

However, we found a positive correlation between the length of the diagnostic delay and both the age at acromegaly diagnosis and the age at the last follow-up. A possible explanation of this finding may be that acromegaly patients with longer diagnostic delay are in this cohort affected more acromegaly-related comorbidities since the time of acromegaly diagnosis. In parallel, patients with more acromegaly related comorbidities were older at the time of last follow-up (as showed in supplementary table 1). These data may suggest that the acromegaly diagnostic delay may cause the occurrence of multiple acromegaly related comorbidities and, as for consequence, the presence of systemic comorbidities may represent a reason for older patients remaining in follow-up. Interestingly, only diagnostic delay and not age-related factors were significantly predicting incident VFs at multivariate analysis. The diagnostic delay in elderly acromegaly patients may be due to the overlap between the features of acromegaly and of the physiological ageing [47]. In a retrospective cohort of 57 newly diagnosed acromegalic patients aged over than 60 years, hypertension, glucose metabolism abnormalities, joint complaints and goiter were the most prevalent comorbidities [48], that typically occur with a high frequency in no acromegaly elderly individuals. In parallel, in this study we found a positive correlation between the diagnostic delay and the age of acromegaly patients at last follow-up.

In this study, we did not find a significant correlation between the occurrence of VFs and the biochemical status of acromegaly and the length of active disease. In this series, in fact, the number of patients with active disease at follow-up was very low, counting only fourteen out of the 172 enrolled patients (8.1%). Similarly, in this cohort of patients at the logistic regression we did not find a significant difference of length of active acromegaly disease among patients with and without incident VFs. Previous data in the literature on the effect of the length of active acromegaly had suggested that a longer period of active acromegaly was associated to an increased risk of incident vertebral fractures [14, 48]. A possible reason of this discrepancy may be due to the shorter duration of active acromegaly in patients with cured/controlled of acromegaly in this cohort. In fact, the large majority of patients reached the cure/control of acromegaly, with a median duration of active disease of 15 months (IQR: 13, range 6–30 months). In previous reports, the duration of active disease ranged from 23 to 186 months [16, 27, 49]*.* As a consequence, the small number of patients with active acromegaly and short duration of active disease in this cohort may explain the absence of correlation with the occurrence of incidental vertebral fractures.

The main limitations of our study are the retrospective design, the low number of patients with active acromegaly disease, the lack of a control elderly population to rule out the impact of aging per se in our population.

In conclusion, our results showed for the first time that the diagnostic delay in acromegaly in a significant cause of skeletal fragility and vertebral fractures. Since these comorbidities are irreversible, our results underline that only a prompt and early diagnosis and treatment of acromegaly may be effective in protecting the patient from its life-long consequences, among which those affecting bone based on our data appear to be particularly time-sensitive.

## Supplementary Information

Below is the link to the electronic supplementary material.

Supplementary file1 (DOCX 12 kb)

## Data Availability

The data presented in this study are available on request from the corresponding author.
