# Supplementary material for: Impact of the diagnostic delay of acromegaly on bone health: data from a real life and long term follow-up experience
Source: Pituitary. 2022 Aug 3;25(6):831–41. doi: 10.1007/s11102-022-01266-4 (PMC9362053; doi:10.1007/s11102-022-01266-4)
Supplement: Supplementary file 1 — Supplementary file1 (DOCX 12 kb) [file 11102_2022_1266_MOESM1_ESM.docx]

Supplementary table 1. Time of diagnostic delay of acromegaly diagnosis and age of last follow-up, according to the number of acromegaly related comorbidities found at the diagnosis of acromegaly. *: median, IQR

| Acromegaly related comorbidities, number | Years of diagnostic delay* | Age at last follow-up* |
| --- | --- | --- |
| Single comorbidity | 7 (7.3) | 49 (13) |
| Two comorbidities | 8 (10) | 55 (14) |
| Three comorbidities | 9 (6) | 58 (12.5) |
| Four comorbidities | 10 (7) | 63 (26) |
| Five comorbidities | 10 (8) | 65 (25.5) |
| Six comorbidities | 15 (8) | 68 (30) |
